# [^161^Tb]Tb-PSMA-617 radioligand therapy in patients with mCRPC: preliminary dosimetry results and intra-individual head-to-head comparison to [^177^Lu]Lu-PSMA-617

**DOI:** 10.7150/thno.92273

**Published:** 2024-02-24

**Authors:** Andrea Schaefer-Schuler, Caroline Burgard, Arne Blickle, Stephan Maus, Christine Petrescu, Sven Petto, Mark Bartholomä, Tobias Stemler, Samer Ezziddin, Florian Rosar

**Affiliations:** Department of Nuclear Medicine, Saarland University, Medical Center, Homburg, Germany.

**Keywords:** ^161^Tb, dosimetry, PSMA, radioligand therapy, mCRPC, prostate cancer

## Abstract

**Rationale:** Evaluation of alternative radionuclides for use in prostate-specific membrane antigen (PSMA)-targeted radioligand therapy (RLT) is currently focusing on ^161^Tb, which may provide advantages by emitting additional Auger and conversion electrons. In this pilot study, we present preliminary dosimetry data for [^161^Tb]Tb-PSMA-617 RLT in a direct comparison with [^177^Lu]Lu-PSMA-617.

**Method:** Six patients with metastatic castration-resistant prostate cancer (mCRPC) underwent treatment with [^177^Lu]Lu-PSMA-617 and subsequently - after inadequate response - with [^161^Tb]Tb-PSMA-617. Whole-body planar and SPECT imaging-based dosimetry of organs at risk (kidneys and salivary glands) and tumor lesions were calculated using IDAC for ^177^Lu and OLINDA/EXM for ^161^Tb. The therapeutic index (TI) of mean tumor-absorbed doses over relevant organs at risk was calculated.

**Results:** Mean absorbed doses to organs at risk of PSMA-RLT were slightly higher for [^161^Tb]Tb-PSMA-617 compared to [^177^Lu]Lu-PSMA-617 (kidneys: 0.643 ± 0.247 vs. 0.545 ± 0.231 Gy/GBq, factor 1.18; parotid gland: 0.367 ± 0.198 vs. 0.329 ± 0.180 Gy/GBq, factor 1.10), but markedly higher regarding tumor lesions (6.10 ± 6.59 vs 2.59 ± 3.30 Gy/GBq, factor 2.40, *p* < 0.001). Consequently, the mean TI was higher for [^161^Tb]Tb-PSMA-617 compared to [^177^Lu]Lu-PSMA-617 for both, the kidneys (11.54 ± 9.74 vs. 5.28 ± 5.13, *p* = 0.002) and the parotid gland (16.77 ± 13.10 vs. 12.51 ± 18.09, *p* = 0.008).

**Conclusion:** In this intra-individual head-to-head pilot study, [^161^Tb]Tb-PSMA-617 delivered higher tumor-absorbed doses and resulted in superior TI compared to [^177^Lu]Lu-PSMA-617. This preliminary data support ^161^Tb as a promising radionuclide for PSMA-RLT in mCRPC.

## Introduction

Prostate cancer continues to rank as the second most prevalent form of malignant disease among men globally in 2022 1. Patients with prostate cancer frequently progress to the state of metastatic castration-resistant prostate cancer (mCRPC) which is unresponsive to physical or chemical castration and is associated with a relatively poor prognosis 2,3. Common treatments for mCRPC patients include new androgen axis drugs as abiraterone or enzalutamide 4,5, chemotherapy with docetaxel or cabazitaxel 6,7, bone seeking radiation therapy with Ra-223 8, and PARP inhibitors as e.g. Olaparib 9. In addition to this treatments, the targeted radioligand therapy (RLT) that specifically targets prostate specific membrane antigen (PSMA) has proven to be a promising approach for reducing total tumor burden and improving survival 10-12. Recently, PSMA-RLT with ^177^Lu was approved by the *FDA* and *EMA* 13,14. Due to its favorable characteristics, the beta-emitting ^177^Lu is currently the most abundantly used radionuclide 15. These properties correspond to a cytotoxicity that frequently achieves a satisfying anti-tumor effect while maintaining a favorable adverse events profile 16-20. However, a fraction of patients still presents to be non- or low responders to PSMA-RLT with ^177^Lu. Therefore, the research for new radionuclides in RLT is ongoing, with the aim to improve PSMA-RLT. Some studies suggested the use of alpha emitters such as ^225^Ac, which showed considerable success, even after non-response to ^177^Lu 21-23. PSMA-RLT with ^225^Ac was used both as monotherapy 21 and as tandem therapy in combination with ^177^Lu 22,23. However, in many cases PSMA-RLT with ^225^Ac showed relevant side effects, especially when applied as monotherapy 21.

A new emerging candidate of interest to clinicians and researchers is ^161^Tb, which has similar chemical properties and physical decay characteristics to the established ^177^Lu 24. Shared characteristics between the two radionuclides include similar mean β-particle energy (^161^Tb: 154 keV vs. ^177^Lu: 133 keV) and γ-ray emission (^161^Tb: 74.6 keV/10.2%; 48.9 keV/17.0% vs. ^177^Lu: 208.4 keV/10.4%; 112.9 keV/6.2%) as well as similar half-life (^161^Tb: 6.906 d vs. ^177^Lu: 6.647 d) 25. However, in contrast to ^177^Lu, ^161^Tb emits a high proportion of conversion and low-energy Auger electrons. These electrons have an ultra-short tissue range (< 500 nm) resulting in a high linear energy transfer (LET) (4 - 26 keV/µm) providing higher local dose densities 26,27. Preclinical studies utilizing cell-based *in vitro* assays have indicated higher efficacy of ^161^Tb over ^177^Lu 27-30, a dose-dependent delay in tumor growth and a prolonged survival in mice treated with [^161^Tb]Tb-PSMA-617 were observed 31. A theoretical study based on clinical data has recently shown that the dose per unit activity in tumor tissue can be increased in RLT by replacing ^177^Lu with ^161^Tb 32. Similar to ^177^Lu, ^161^Tb can also be produced in a nuclear reactor. Although initially less available than ^177^Lu, there is increasing commercial availability of ^161^Tb reaching high level of specific activity and chemical purity. However, experience with ^161^Tb from clinical trials and data derived from real-world settings remain scarce. Only one feasibility study with application of [^161^Tb]Tb-DOTATOC in 2 patients with neuroendocrine neoplasms and two case reports of PSMA-RLT with [^161^Tb]Tb-PSMA-617 have been published yet, all showing promising results 33-35. A clinical phase I/II study at the Peter MacCallum Cancer Centre, Australia (VIOLET; NCT05521412) is currently underway recruiting patients for PSMA-RLT with [^161^Tb]Tb-PSMA-I&T.

In this pilot study, we present a preliminary dosimetry analysis investigating the potential of [^161^Tb]Tb-PSMA-617 for RLT in patients with mCRPC. The dosimetric evaluation of [^161^Tb]Tb-PSMA-617 RLT was performed in an intra-individual head-to-head comparison with [^177^Lu]Lu-PSMA-617.

## Material and Methods

### Patients and study objective

In total, n = 6 consecutive mCRPC patients enrolled in a prospective register (REALITY study, NCT 04833517) were included in this study receiving [^161^Tb]Tb-PSMA-617 between September 2022 and February 2023. The cohort was heavily pretreated with androgen-deprivation therapy (ADT), new androgen axis drugs (NAAD) and chemotherapy. All patients underwent PSMA-RLT with conventional radionuclides, ^177^Lu and ^225^Ac prior starting [^161^Tb]Tb-PSMA-617 RLT and experienced progression or inadequate response. Detailed patients characteristics including age, blood values as e.g. PSA serum value, *Eastern Cooperative Oncology Group* (ECOG) performance status, sites of metastases and prior therapies are compiled in table 1. All patients received 1 cycle of [^161^Tb]Tb-PSMA-617. The mean administered activity of [^161^Tb]Tb-PSMA-617 was 6.4 ± 1.2 GBq (range 5.1 - 8.7 GBq). The administration of [^161^Tb]Tb-PSMA-617 was applied on a compassionate use basis, following the regulations of the German Pharmaceutical Act §13 (2b). The applying physician took accountability for medical indication and labeling of the tracer. All patients provided written consent to the study's terms and conditions after being comprehensively informed about the general risks associated with the administration of novel therapy substances, radiation exposure and possible adverse effects. All patients gave consent to the publication of data resulting from the study, in accordance with the guidelines of the declaration of Helsinki. All 6 patients were previously treated with [^177^Lu]Lu-PSMA-617 and 4/6 patients have received [^225^Ac]Ac-PSMA-617 as augmentation to [^177^Lu]Lu-PSMA-617 RLT. The last cycle was performed 2-4 months prior to [^161^Tb]Tb-PSMA-617 RLT with a mean administered activity of 6.9 ± 1.2 GBq (range 4.2 - 8.0 GBq) [^177^Lu]Lu-PSMA-617. During [^177^Lu]Lu-PSMA-617 and [^161^Tb]Tb-PSMA-617 RLT, antihormonal treatment with ADT and NAAD were continued unchanged to avoid altering PSMA expression and biasing the treatment effect 36,37.

The objective of this study was to calculate estimates of absorbed doses of target tumor lesions and organs for [^161^Tb]Tb-PSMA-617 and to compare these estimates with those for [^177^Lu]Lu-PSMA-617 from the previous cycle.

### Radiolabeling and quality control

The radiolabeling of PSMA-617 with ^161^Tb or ^177^Lu and the quality control of both, [^161^Tb]Tb-PSMA-617 and [^177^Lu]Lu-PSMA-617, were accomplished in analogy to the methodology published previously for [^177^Lu]Lu-PSMA-617 38. For a typical labeling of [^161^Tb]Tb-PSMA-617, ^161^Tb ([^161^Tb]TbCl_3_ in 0.05 M HCl, TERTHERA B.V., Breda, Netherlands) was added to sodium acetate buffer (1.0 M, pH 4.5) containing PSMA-617 (Advanced Biochemical Compounds, ABX GmbH, Radeberg, Germany) to reach a specific activity of ~42 MBq/nmol. The reaction mixture (pH 4.5) was incubated for 25 min at 95 °C. A quality control per reversed-phase high-performance liquid chromatography (Shimadzu LC-20AT high pressure liquid chromatography (HPLC) system) was performed. The radiochemical yields and purities of both radiotracers were ≥ 99%.

### Imaging

For dosimetric calculations, scintigraphy was performed after injection of [^161^Tb]Tb-PSMA-617. Scintigraphic images were acquired on a hybrid scanner, the Philips BrightView XCT (Philips Medical Systems, Hamburg, Germany). The scanner was equipped with low-energy, high resolution (LEHR) parallel-hole collimators for imaging ^161^Tb and with medium-energy collimators for imaging ^177^Lu. For imaging with ^161^Tb an uniformity correction map was acquired as described in the supplemental data. On days 1, 2, and 4 after injection (approximately 24 hours, 48 hours, and at least 96 hours post-injection), planar whole-body images were acquired. On day 1 after injection, a SPECT/CT scan of the head and neck (“head and neck SPECT/CT”) was performed. Additionally, on day 2 post injection, another SPECT/CT scan, from the liver down to the pelvis (“abdomen-SPECT/CT”), was acquired.

The energy windows for ^161^Tb and ^177^Lu imaging were set to 74.6 keV and 208 keV, respectively, with a width of 20% as proposed by MIRD and used in previous studies on ^177^Lu imaging 39,40. In order to perform scatter correction on the whole-body images based on the dual-energy window technique (DEW) 39, respective low scatter windows were set at 69.4 keV for ^161^Tb and at 187 keV for ^177^Lu, both with a width of 15% 39. Other image protocol parameters applied for both radionuclides were as follows: the scanning speed for whole body acquisition was 15 cm/min on day 1 and day 2 and 12 cm/min on day 4. The matrix size was 256 × 1024, and the pixel size 4.66 × 4.66 mm^2^. Prior to administering the radiopharmaceutical, a whole-body blank scan and a whole-body transmission scan of the patient were acquired using a flat phantom filled with aqueous solution of ^161^Tb and ^177^Lu, respectively. The corresponding images were used for attenuation correction of the planar whole-body data. For both radionuclides, 60 SPECT projections over 360° projections were acquired with a frame-time duration of 20 s for head and neck and a frame-time of 30 s for abdomen SPECT, respectively. The matrix size was 128 × 128, and the pixel size 4.66 × 4.66 mm^2^. CT images were acquired in low-dose technique using an X-ray tube voltage of 120 keV and a tube current of 10 mA. The matrix and the pixel size were 256 × 256 and 2.33 × 2.33 mm^2^, respectively. CT data was applied to calculate an attenuation map at 100 keV, which was then converted to the respective emission energies and applied for attenuation and scatter correction of SPECT data 41. Iterative SPECT image reconstruction parameters were chosen based on the recommendations proposed in the MIRD pamphlets 23 and 26 42,43. An iterative 3D-ordered subset expectation maximization (OSEM) algorithm was applied employing 3 iterations, 8 subsets, Butterworth filtering (0.5, 10th order), and a slice thickness of 4.66 mm. CT images were converted to a matrix with a pixel size of 128 × 128 and 4.66 × 4.66 mm^2^ and then fused with the SPECT slices.

### Radiation dosimetry

Dosimetry calculations for both radionuclides, ^161^Tb and ^177^Lu, were based on the MIRD scheme and included the following tissues: kidneys, liver, salivary glands (parotid gland and submandibular gland) and tumor lesions. According to the MIRD dosimetric approach the time-dependent activity of the organs and lesions need to be determined. This evaluation comprises delineation of corresponding region-of-interest (ROI) and volume-of-interest (VOI) and calculation of the proportionate injected activities [%] for each time point 44. The applied software packages were QDOSE (ABX-CRO, Dresden, Germany) and OLINDA/EXM version 1.0 45. The dosimetric workflow established for ^177^Lu 46 was applied analogously for ^161^Tb. Planar whole-body images were quantified on a pixel-by-pixel basis, using the conjugate-view method 39. To convert count rate to activity for planar scintigraphy respective calibration factors were used as described in supplemental data. All whole-body images were co-registered using non-rigid registration methods. Boundary ROI enclosing the organs as well as background ROI close to the respective organs or tumor lesions were manually drawn in the whole-body images of the first time point of the ^177^Lu scintigraphy and then copied on to the planar whole-body images of the other time points and the planar whole-body scintigrams of ^161^Tb. Tumor lesions (bone metastases or lymph node metastases) presenting no overlap with tissues of high uptake were selected for analysis exclusively. Attenuation correction was performed according to MIRD 39 using the respective blank and the respective patient whole body transmission scan thereby taking into account the body and organ thicknesses of the patient determined by CT. Background correction included determination of the activity per pixel in the respective background ROI and subsequently scaling to the number of pixels in the organ ROI. Delineation of the organ within the manually drawn boundary ROI on both the anterior and the posterior image was performed by an experienced physicist and the responsible physician. For this purpose, a threshold-based segmentation was applied. The planar-derived time-activity curves were created by mono-exponential regression of the serially measured activities using weighted least squares method.

In the SPECT/CT images, boundary VOI were manually drawn which enclosed the source organs avoiding neighboring structures. Volume and activity estimation were performed within the boundary using a fuzzy locally adaptive Bayesian (FLAB) segmentation algorithm for automatic volume delineation 47. The measured voxel values were converted to activity using the camera calibration factor and then corrected by applying the respective volume-dependent recovery coefficients (compare supplemental data, Table S1) and combined with the time-activity data from the planar images. Here, SPECT activity estimation was used to rescale the planar-derived time-activity curve 39,42,46.

The rescaled time-activity curve was fitted by monoexponential regression and the time-integrated activities (TIA) in the source regions determined as the area under the curve by analytical integration and used to calculate the respective time-integrated activity coefficients (TIAC) 46. For [^177^Lu]Lu-PSMA-617, this last step was performed in QDOSE whereas for [^161^Tb]Tb-PSMA-617, the rescaled activity data was imported into the software OLINDA/EXM 45 to perform the respective calculations. Here, the absorbed doses to the tumor lesions and the salivary glands were calculated using the spheres model and the respective reference man for the kidneys and the liver. Estimation of the absorbed dose for [^177^Lu]Lu-PSMA-617 was performed using the IDAC 2.1 software implemented in QDOSE 48. Here, the IDAC reference man was applied for the kidneys and the liver and the spheres model for the salivary glands and the tumor lesions, respectively.

The volumes of the tumor lesions were determined from the pre-therapeutic ^68^Ga-PSMA-11 PET/CT images. For this purpose, a volume-of-interest with an isocontour of 20-40% of SUVmax was drawn using a dedicated workstation (Syngo. Via, Siemens Healthcare, Erlangen, Germany). Organ masses for the salivary glands were taken from *International Commission on Radiological Protection (ICRP)* publication 89 with 25 g estimated weight for the parotid and 12.5 g for the submandibular gland 49.

### Individual Therapeutic Index (TI) and relative Therapeutic Index (rTI)

To evaluate the potential therapeutic effect of the radiopharmaceutical PSMA-617 in relation to organs at risk when labeled with both, the radionuclides ^161^Tb and ^177^Lu, the individual therapeutic index (TI) was applied 50. TI was defined by calculating the mean radiation dose absorbed in tumor lesions divided by the mean radiation dose delivered to a relevant organ at risk. As in the case of PSMA-RLT the relevant organs at risk are known to be the kidneys and the salivary glands 10,17, the TI for the kidneys (TI-kidney) and exemplarily for the salivary glands, the TI for the parotid gland (TI-parotid) were determined. Furthermore, the ratio of the TI for [^161^Tb]Tb-PSMA-617 to the TI for [^177^Lu]Lu-PSMA-617 was calculated for each organ at risk and reported as relative therapeutic index (rTI-kidney and rTI-parotid). A rTI value > 1 is considered as an indication of favorable energy deposition by [^161^Tb]Tb-PSMA-617.

### Patient-based outcome and adverse events

To evaluate the efficacy of one cycle of [^161^Tb]Tb-PSMA-617, serum PSA was measured at baseline and 5 ± 2 weeks after the [^161^Tb]Tb-PSMA-617 RLT. Relative PSA change was calculated to assess biochemical response using the *Prostate Cancer Working Group 3 (PCWG 3)* criteria 51. Blood tests including hemoglobin, leukocytes and platelets, and creatine-based estimated glomerular filtration rate (GFR) were performed to assess safety profile and adverse events. In addition, xerostomia was assessed using a dedicated questionnaire. Adverse events were graded according to the *Common Terminology Criteria for Adverse Events (CTCAE)* version 5.0. Furthermore, change in pain rated by visual analog scale (VAS) ranging from 0 - 10 was analyzed as an additional marker for quality of life.

### Statistical Analysis

All continuous data reported is expressed as mean ± standard deviation and range. A paired non-parametric Wilcoxon matched-pairs signed rank test was performed to compare absorbed lesion doses. Statistical analyses were conducted using GraphPad Prism (GraphPad Software; Version 8.0, San Diego, USA).

## Results

### Dosimetry of normal organs after administration of [^161^Tb]Tb-PSMA-617

**Figure 1:**
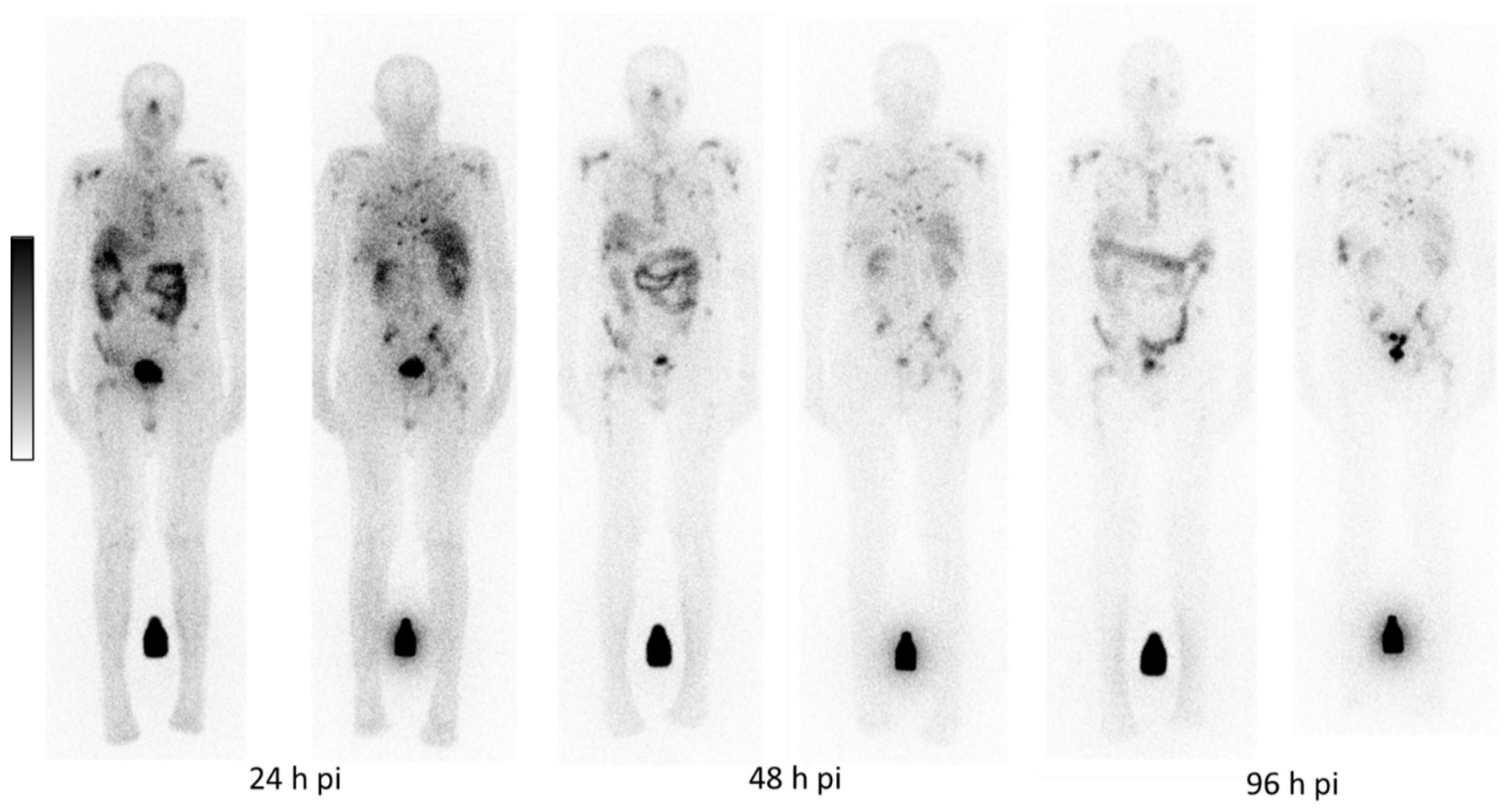
Representative example of whole-body scintigrams acquired at three different time-points post administration of [^161^Tb]Tb-PSMA-617.

Physiological uptake was observed in the salivary and lacrimal glands, kidneys, liver, intestine and urinary tract. Figure 1 depicts a representative example of [^161^Tb]Tb-PSMA-617 planar whole-body scintigraphy demonstrating long-term retention of [^161^Tb]Tb-PSMA-617 in the metastases with relatively low residual uptake in normal organs.

The monoexponential curve-fitting parameters, the time-integrated activity coefficients (TIAC) for each source organ and the respective estimated absorbed doses according to OLINA/EXM are summarized in Table 2. Among the normal tissues, the liver received the lowest (0.148 ± 0.080 Gy/GBq) whereas the kidneys received the highest absorbed doses for [^161^Tb]Tb-PSMA-617 with 0.643 ± 0.247 Gy/GBq, followed by the salivary glands (submandibular gland: 0.372 ± 0.188 Gy/GBq; parotid gland: 0.367 ± 0.198 Gy/GBq). In comparison, the estimated absorbed doses in normal organs post administration of [^177^Lu]Lu-PSMA-617 are also presented in Table 2.

**Figure 2:**
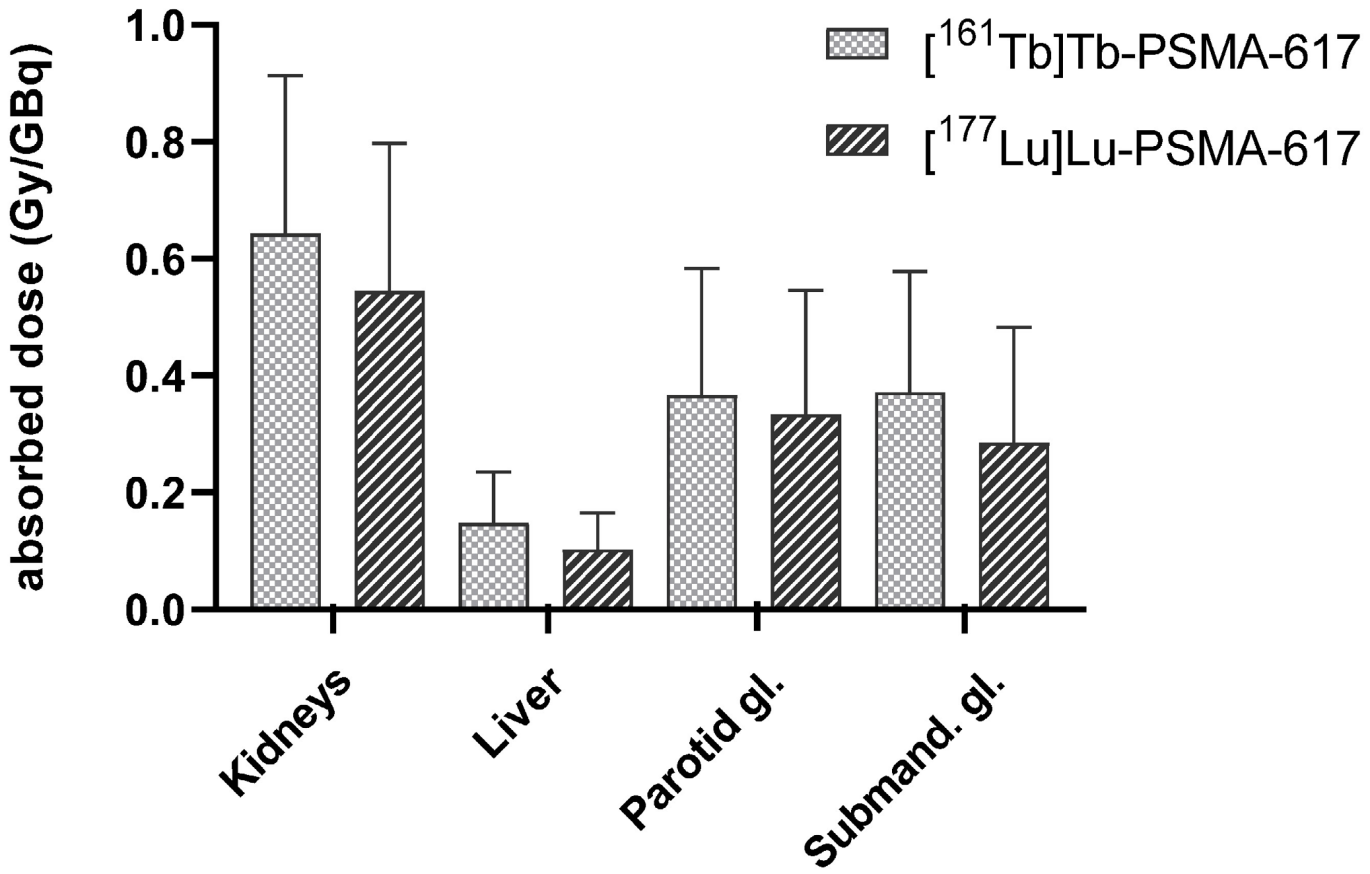
Mean absorbed doses (Gy/GBq) delivered to kidneys, liver, parotid and submandibular glands determined for [^161^Tb]Tb-PSMA-617 and [^177^Lu]Lu-PSMA-617 over all patients.

Figure 2 shows the mean estimated absorbed doses in normal organs post administration of [^161^Tb]Tb-PSMA-617 vs. [^177^Lu]Lu-PSMA-617 over all patients and Table 3 presents the respective individual organ doses. In comparison, the respective monoexponential curve-fitting parameters and time-integrated activity coefficients (TIAC) for [^177^Lu]Lu-PSMA-617 are presented in the supplemental data (Table S2 ). In order to present TIAC of kidney and liver in relation to organ function parameters, corresponding data are also provided in the supplemental data (Table S3).

### Dosimetry of Tumor Lesions

**Figure 3:**
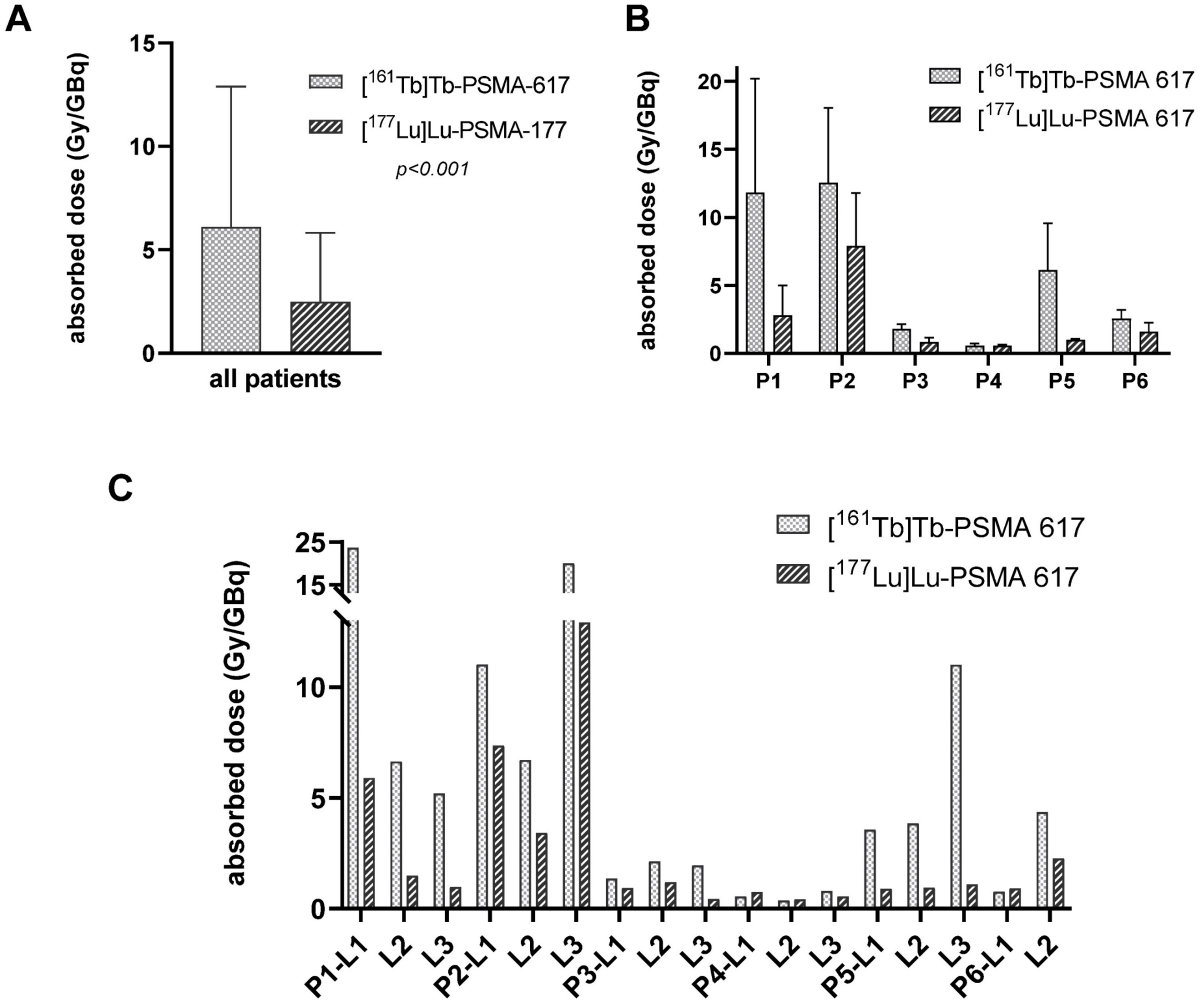
Absorbed doses in tumor lesions determined after application of [^161^Tb]Tb-PSMA 617 and [^177^Lu]Lu-PSMA 617, respectively. **A**: for all patients and lesions, **B**: mean tumor lesion absorbed dose per patient,** C**: absorbed dose in each individual tumor lesion.

A total of n = 17 representative tumor lesions were analyzed. Localization and size of the lesions were summarized in supplemental data (Table S4). The mean absorbed dose of the tumor lesions was 6.10 ± 6.59 Gy/GBq (range: 0.55 - 23.63 Gy/GBq) for [^161^Tb]Tb-PSMA-617 and 2.59 ± 3.30 Gy/GBq (range: 0.42 - 12.91 Gy/GBq) for [^177^Lu]Lu-PSMA-617 (*p* < 0.001). In addition, the effective half-life in tumor lesions was longer for [^161^Tb]Tb-PSMA-617 (mean half-life 46.1 ± 19.2 h) than for [^177^Lu]Lu-PSMA-617 (mean half-life 35.3 ± 6.3 h, *p* = 0.006). Considering each individual patient, the mean absorbed dose of the lesions was higher for [^161^Tb]Tb-PSMA-617 than for [^177^Lu]Lu-PSMA-617 (compare Figure 3B). On a lesion-based analysis 14/17 lesions (82.4%) showed a higher absorbed dose for [^161^Tb]Tb-PSMA-617 than for [^177^Lu]Lu-PSMA-617 and another 2/17 lesions showed very close values for both kind of RLT (*p* < 0,001, Figure 3C).

### Therapeutic Index TI and relative Therapeutic Index rTI

**Figure 5:**
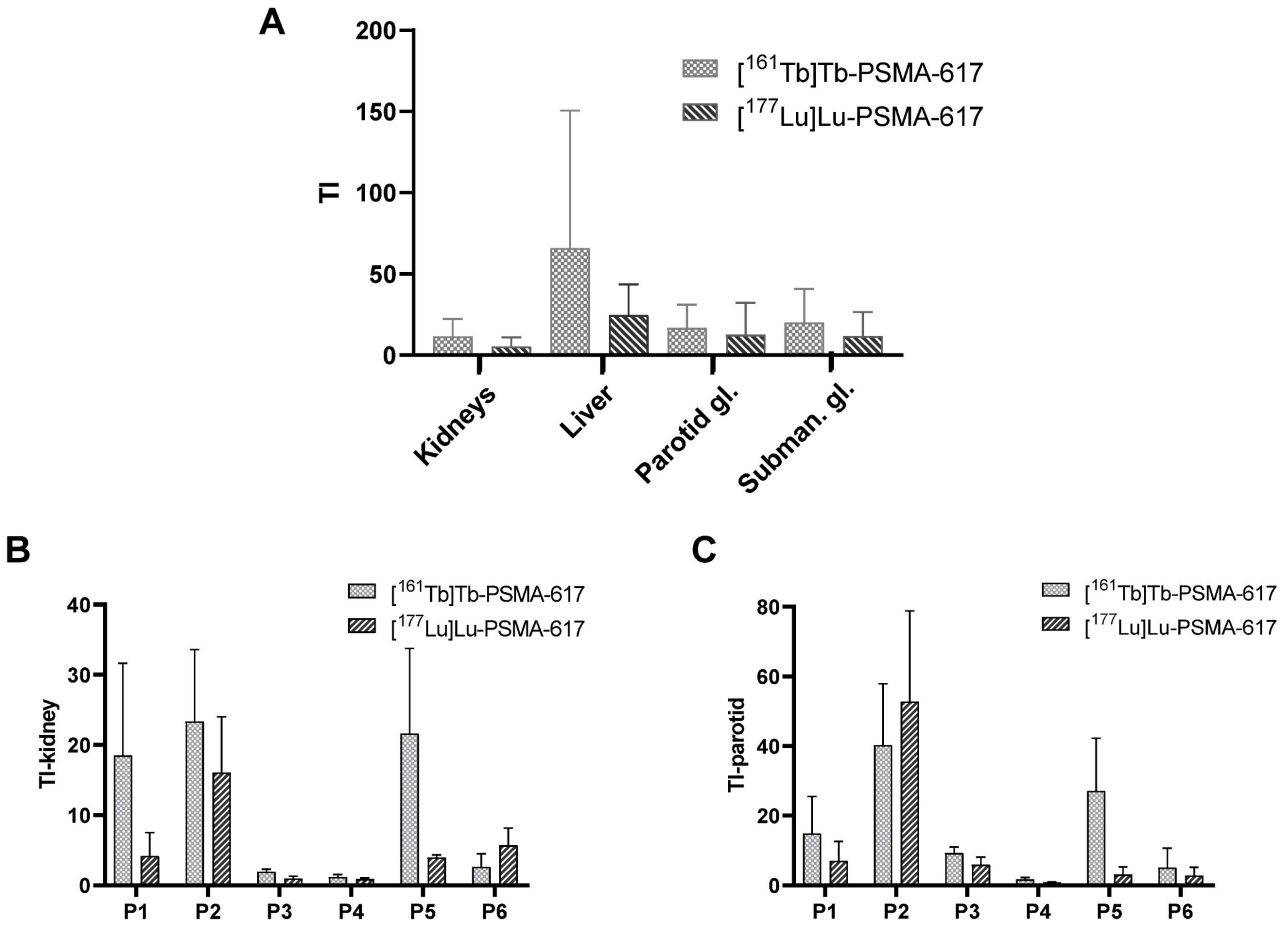
** A**: Therapeutic index (TI) over kidneys, liver, parotid gland and submandibular gland determined for [^161^Tb]Tb-PSMA-617 and [^177^Lu]Lu-PSMA-617. **B**: TI-kidney for each individual patient, **C**: TI-parotid for each individual patient.

**Figure 6:**
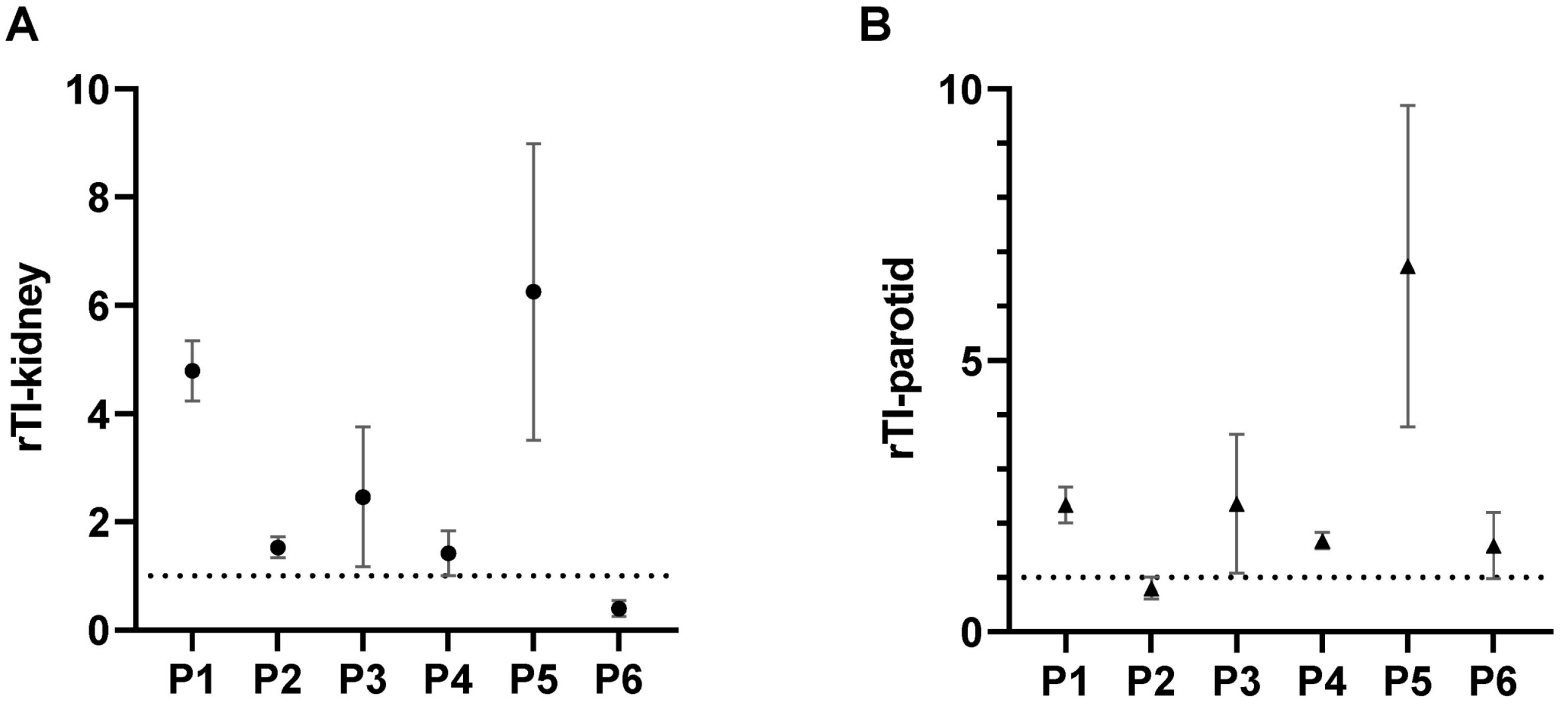
Relative therapeutic index (rTI) for each individual patient. **A:** rTI-kidney and **B:** rTI-parotid gland. Values of rTI > 1 indicate favorable dose distribution for [^161^Tb]Tb-PSMA-617 compared with [^177^Lu]Lu-PSMA-617 and vice versa. For both organs, kidneys and parotid gland, 5/6 patients had favorable distribution of [^161^Tb]Tb-PSMA-617 and 1/6 patient had favorable distribution of [^177^Lu]Lu-PSMA-617.

The mean TI for the kidneys, the liver and the salivary glands are presented in Figure 5A, comparing [^161^Tb]Tb-PSMA-617 with [^177^Lu]Lu-PSMA-617. For the kidneys as reference organ-at-risk, the mean TI-kidney were 11.54 ± 9.74 for [^161^Tb]Tb-PSMA-617 and 5.28 ± 5.13 for [^177^Lu]Lu-PSMA-617 (*p* = 0.002). Intra-individual comparison of [^161^Tb]Tb-PSMA 617 and [^177^Lu]Lu-PSMA-617 revealed higher TI-kidney in 5/6 (83.6%) patients for [^161^Tb]Tb-PSMA-617 and consequently, rTI-kidneys > 1.0 was observed. The patient with a higher TI-kidney for [^177^Lu]Lu-PSMA-617 showed values of 5.69 for [^177^Lu]Lu-PSMA-617 vs. 2.63 for [^161^Tb]Tb-PSMA-617. Consequently, the rTI-kidney was less than 1 for this patient (rTI-kidney: 0.4) compared to a mean rTI-kidney of 2.96 ± 1.68 for the other 5/6 patients. The patient individual TI-kidney and rTI-kidney are presented in Fig. 5B and 6A, respectively.

Similar results were observed for the parotid glands. Here, mean TI-parotid of 16.77 ± 13.10 for [^161^Tb]Tb-PSMA-617 compared with 12.51 ± 18.09 for [^177^Lu]Lu-PSMA-617 were observed (*p* = 0.008). Intra-individual comparisons of [^161^Tb]Tb-PSMA-617 and [^177^Lu]Lu-PSMA-617 showed higher TI-parotid in 5 out of 6 patients (83.4%) and consequently rTI-parotid > 1 (mean rTI: 2.71 ± 1.62). In 1 patient, [^177^Lu]Lu-PSMA-617 showed a higher TI-parotid (TI-parotid: 52.3 vs 40.2, respectively) and consequently a rTI-parotid < 1 (rTI-parotid: 0.80). The individual TI-parotid and rTI-parotid are presented in Fig. 5C and 6B, respectively.

### Patient-based outcome and adverse events

After the applied one cycle of [^161^Tb]Tb-PSMA-617, three patients experienced a decrease of PSA value by 53.4%, 24.2% and 18.6%, respectively. In the remaining three patients the PSA value increased by 18.0%, 48.6% and 73.2%, respectively. Patient individual PSA values and change are summarized in the supplemental data (Table S5). Based on biochemical response assessment by PCWG 3 criteria with defining progressive disease (PD) as PSA increase > 25%, partial remission (PR) as PSA decrease > 50%, and stable disease in any other case: 1 patient showed PR, 3 patients SD, and 2 patients PD. The patient with PR and one of the patients with SD were matched with the two patients who had received only [^177^Lu]Lu-PSMA-617 monotherapy in the past. The remaining 2 patients with SD and 2 patients with PD had been previously augmented with [^225^Ac]Ac-PSMA-617. After one cycle of [^161^Tb]Tb-PSMA-617, the glomerular filtration rate (GRF) was mainly stable, with only one patient experiencing an increase in CTCAE (from 0 to 1). Furthermore, only one patient experienced a relevant decrease in hemoglobin, leukocytes, and platelets with an increase in CTCAE from 2 to 3, 1 to 2, and 1 to 3, respectively. However, this increase in anemia, leukocytopenia, and thrombocytopenia was more likely due to tumor progression (ΔPSA +72.3% and increasing bone marrow involvement). Increasing xerostomia was observed in two patients (CTCAE from 0 to 1 and 1 to 2), but these two patients were pretreated with ^225^Ac. In addition, a reduction in pain (as indicated by a decrease in VAS from 8 to 7 and 2 to 1) was observed in two patients; only one patient experienced an increase in pain (VAS from 2 to 3).

## Discussion

In this pilot study, we present first data on therapeutic radiation dosimetry regarding normal organs and tumor lesions for [^161^Tb]Tb-PSMA-617 compared to [^177^Lu]Lu-PSMA-617 in 6 patients with mCRPC. Comparing the biodistribution of [^161^Tb]Tb-PSMA-617 to that of [^177^Lu]Lu-PSMA-617, a similar distribution pattern was observed over time with intense uptake of radiolabeled PSMA ligands in salivary glands, kidneys, liver, and small intestine and a high retention in tumor lesions. Quantitative results in this cohort of patients indicate that [^161^Tb]Tb-PSMA-617 provides notably higher absorbed dose to tumor lesions in comparison to [^177^Lu]Lu-PSMA-617. Furthermore, mean absorbed doses to the relevant organs at risk of PSMA-RLT were only slightly higher for [^161^Tb]Tb-PSMA-617 compared to [^177^Lu]Lu-PSMA-617.

Our data reveals that [^161^Tb]Tb-PSMA-617 delivered a radiation dose to tumor lesions that was, on average, 2.4 times higher than the radiation dose from [^177^Lu]Lu-PSMA-617. In our cohort, a mean tumor lesion dose of 6.10 ± 6.59 Gy/GBq (range: 0.55 - 23.63 Gy/GBq) was observed for [^161^Tb]Tb-PSMA-617 compared to 2.59 ± 3.30 Gy/GBq (range: 0.42 - 12.91 Gy/GBq) for [^177^Lu]Lu-PSMA-617. Although absorbed tumor doses varied by patient as well as by lesion, our pilot study allows a first comparison between the two radiopharmaceuticals. Relatively high inter-patient variability of absorbed doses in tumor lesions has previously also been reported in several studies for [^177^Lu]Lu-PSMA-617 as well as for other PSMA ligands as e.g. [^177^Lu]Lu-PSMA I&T 50,52,53. Exemplarily for [^177^Lu]Lu-PSMA-617, Violet et al. 52 reported mean absorbed doses of 5.28 Gy/GBq (range: 0.41-10.71Gy/GBq) for bone metastases and of 3.91 Gy/GBq (range: 0.52-16.23 Gy/GBq) for lymph node metastases whereas Okamoto et al. 53 reported a mean absorbed dose for all metastases of 3.2 ± 2.6 Gy/GBq (range: 0.22 - 12.03 Gy/GBq) for [^177^Lu]Lu-PSMA I&T. In comparison to this data, the radiation dose delivered by [^161^Tb]Tb-PSMA-617 across the tumor lesions was higher than that reported for the different [^177^Lu]Lu-PSMA-ligands. This could be of clinical relevance as it has already been reported for [^177^Lu]Lu-PSMA-617, that a higher radiation dose in the tumor achieves improved efficacy 52.

The high tumor doses delivered by ^161^Tb might be explained by the physical decay characteristics of ^161^Tb co-emitting a high proportion of conversion and low-energy Auger electrons. The benefit of those electrons has previously been demonstrated by several groups applying Monte Carlo simulation on ^161^Tb to assess electron doses and distribution 26,27. The work of these groups demonstrated that ^161^Tb is a radionuclide particularly appropriate for irradiation of micro-metastases due to the short range of these electrons and therefore, at least in the context of micro-metastases, ^161^Tb seems to hold decisive advantages over ^177^Lu. In addition, recently published studies using *in vitro* methods and mice models confirm the beneficial effects of RLT with ^161^Tb and an enhanced therapeutic efficacy of ^161^Tb over ^177^Lu 29,30. Exemplarily, it was demonstrated that the exposure to [^161^Tb]Tb-PSMA-617 in comparison to [^177^Lu]Lu-PSMA-617 reduced both the viability and the survival of PC-3 PIP tumor cells. Moreover, in PC-3 PIP tumor bearing mice treated with [^161^Tb]Tb-PSMA-617 an improved inhibition of tumor growth as well as prolonged survival of tumor affected animals was observed 29. In addition, a recently published theoretical paper by Verburg et al. showed that ^161^Tb, due to its physical properties, delivers a higher absorbed dose to tumor lesions than ^177^Lu by a theoretical factor of about 1.4 if an identical biodistribution is considered 32. The factor of 2.4 estimated in our pilot study, therefore allows the assumption that a biological component must also be taken into account.

Besides the high absorbed doses of [^161^Tb]Tb-PSMA-617 in tumor lesions, a low physiological uptake by normal organs is a required property to use this radionuclide in RLT. With respect to PSMA-RLT, the kidneys and in addition the salivary glands are usually regarded as dose-limiting organs at risk 10,17. It was observed, that the radiation dose to the kidneys may increase the risk of late renal function impairment, especially in late-stage mCRPC patients 54. The VISION study reported that also dry mouth was one of the most common adverse events for [^177^Lu]Lu-PSMA-617 RLT 10. In our cohort, the mean absorbed doses delivered to the relevant organs at risk of PSMA-RLT were higher for [^161^Tb]Tb-PSMA-617 compared to [^177^Lu]Lu-PSMA-617 but only by a factor of 1.18 for the kidneys (0.643 ± 0.247 Gy/GBq vs. 0.545 ± 0.231 Gy/GBq) and of 1.10 for the parotid glands (0.367 ± 0.198 Gy/GBq vs. 0.329 ± 0.180 Gy/GBq). Although the renal doses in our cohort vary from patient to patient (range: 0.283 - 0.974 mGy/MBq), they are in line with the wide range of values that has been reported previously in several studies for [^177^Lu]Lu-PSMA-617 (ranging from 0.4 ± 0.2 Gy/GBq to 0.8 ± 0.3 Gy/GBq) 52,55. To compare the therapeutic efficacy of RLT with [^161^Tb]Tb-PSMA-617 and [^177^Lu]Lu-PSMA-617 the concept of TI as proposed by Feuerecker et al was used 50. Notably, the mean TI-kidney [^161^Tb]Tb-PSMA-617 was 2.19 times higher than that of [^177^Lu]Lu-PSMA-617, and the corresponding rTI-kidney favored ^161^Tb in 5 of 6 patients, indicating a clear advantage of [^161^Tb]Tb-PSMA-617 RLT over [^177^Lu]Lu-PSMA-617 RLT. This result appears to be clinically relevant since higher observed TI might allow, by adjusting the administered activity accordingly, to either I) increase the dose delivered to the tumor while the organs receive the equivalent dose compared to ^177^Lu, or II) decrease the dose to the organs thereby reducing the risk of adverse events while achieving an equivalent dose to the tumor.

Regarding the salivary glands, our values of absorbed doses for [^161^Tb]Tb-PSMA-617 as well as for [^177^Lu]Lu-PSMA-617 are lower than the values previously published by others. Several authors reported mean absorbed doses ranging from 0.58 to 1.41 Gy/GBq for [^177^Lu]Lu-PSMA-617 46,52,55, and similar values for [^177^Lu]Lu-PSMA-I&T (ranging from 0.55 to 0.8 Gy/GBq) 50,53. This may probably be explained by the intense pretreatment of the patients in our cohort, including PSMA-RLT with multiple cycles of ^177^Lu as well as ^225^Ac labeled ligands, potentially providing particular impact on the salivary glands 21. Two patients in our cohort experienced worsening of xerostomia (from grade 0 to 1 and 1 to 2), one of the most common adverse events of RLT. The VISION study reported grade 1 / 2 xerostomia in 38.8% patients, the REALITY study in 20.9% patients receiving [^177^Lu]Lu-PSMA-617 10,16. In comparison, higher frequency of xerostomia was observed after alpha-emitting [^225^Ac]Ac-PSMA-617 treatment, e.g. of 85% reported by Sathekge et al. and of 100% by Feuerecker et al. 21,56. As in the latter study the patients were pretreated by [^177^Lu]Lu-PSMA-617, the increased frequency was explained by the cumulative toxicity of both [^177^Lu]Lu-PSMA-617 and [^225^Ac]Ac-PSMA-617 RLT. The two patients of our cohort had also already been intensively pretreated with ^177^Lu as well as with ^225^Ac, so that the xerostomia was probably caused by the intensive cumulative irradiation including alpha radiation and not clearly attributable to one cycle of ^161^Tb. Moreover, the calculated TI for the salivary glands in our cohort were 1.34 (parotid gland) and 1.70 (submandibular gland) times higher for [^161^Tb]Tb-PSMA-617 than for [^177^Lu]Lu-PSMA-617. Like the rTI-kidney, the rTI-parotid favored [^161^Tb]Tb-PSMA-617 in 5 of 6 patients, suggesting an improved ratio of energy deposition in the tumor lesions compared to the parotid glands.

Based on the demonstrated dosimetric results, ^161^Tb reveals superiority over ^177^Lu in terms of absorbed tumor dose and thus potential antitumor effect. Consequently, there might be a potential opportunity for patients progressing on ^177^Lu- based PSMA-RLT to benefit from a switch to ^161^Tb-based treatment, as may be supported by the data of the two patients pretreated exclusively with multiple cycles of [^177^Lu]Lu-PSMA-617. After one cycle of [^161^Tb]Tb-PSMA-617, one patient showed PR and the other SD. However, in contrast, PSMA RLT with ^161^Tb does not seem to be a promising option in patients previously not responding adequately to [^225^Ac]Ac-PSMA-617; in our 4 patients with this mentioned condition, no significant imaging response or PSA response could be observed. Future studies to investigate [^161^Tb]Tb-PSMA-617 RLT in patients with inadequate response to [^177^Lu]Lu-PSMA-617 RLT seem to be of particular interest. In addition, the use of ^161^Tb as an alternative to ^177^Lu when initiating PSMA RLT could be a focus of future trials.

Our pilot study has certain limitations. First, the study suffers from its retrospective nature and the small cohort of patients. Further studies are necessary, involving a larger number of patients to confirm the results and enable more detailed analyses. The results and comparison may be biased by the time between the two compared cycles, in particular by therapy-related changes or a possible tumor progression. The present study does not determine the clinical impact of higher tumor-absorbed radiation doses delivered by ^161^Tb, but might serve as a rational starting point for future studies. Furthermore, adverse events were only analyzed in short term after the cycle of [^161^Tb]Tb-PSMA-617 RLT. Generally, using a new radionuclide or radioligand, red bone marrow dosing is an essential issue. However, calculation of the dose in the bone marrow based on scintigraphic imaging would have been error-prone for the present cohort, since the majority of the patients showed multiple tumor lesions in the trunk skeleton. In addition, due to the retrospective character no blood samples for dosimetric calculations were available. It should be noted that in this pilot study we concentrated on the dosimetry of the organs at risk; in future studies other organs such as the intestine or spleen should also be evaluated. Moreover, there are some challenges in dosimetric imaging of ^161^Tb. The energies of photons emitted by ^161^Tb are all at low energies resulting in a higher scatter fraction and increased attenuation compared to ^177^Lu photons. For this reason, additional uniformity correction was needed as a minimum prerequisite for imaging with ^161^Tb. To further improve SPECT image quality with ^161^Tb by reducing noise level and increasing contrast, the use of Monte Carlo-based reconstruction algorithm could be taken into account 57. However, to date such reconstruction algorithms are rarely available, especially in commercial systems. Furthermore, the clinical value of images obtained with those algorithms has yet to be proven. In addition, patient comfort and management allowed planar scintigraphy only at 3 time points and only one SPECT/CT imaging per day. However, several studies have shown that dosimetric estimation based on this scintigraphy concept is reliable for PSMA-RLT and PRRT 46,55,58,59.

## Conclusion

This intra-individual head-to-head pilot study reveals that [^161^Tb]Tb-PSMA-617 delivers markedly higher tumor-absorbed doses compared to [^177^Lu]Lu-PSMA-617, whereas the absorbed doses of the relevant organs at risk were only slightly higher. In particular, superior TI-kidney and TI-parotid were observed for [^161^Tb]Tb-PSMA-617 in 5/6 patients. This supports ^161^Tb as a promising candidate for use as radionuclide in PSMA-RLT. Further studies with larger patient cohorts, ideally in a prospective setting, are recommended to confirm this observation.

## Supplementary Material

Supplementary materials and methods, tables.

## Figures and Tables

**Table 1:** Patient characteristics

Patient characteristics	Value
**Age**	
Median (range)	74 (69 - 85)
**PSA,** in [ng/mL]	
Median (range)	280 (44 - 2148)
**ALP,** in [U/L]	
Median (range)	91 (24 - 195)
**Hemoglobin,** in [g/dL]	
Median (range)	9 (8-14)
< 13 g/dL, n (%)	5 (83.3)
**GFR,** in [mL/min]	
Median (range)	56 (32-91)
< 60 mL/min, n (%)	4 (66.7)
**GOT**, in [U/L]	
Median (range)	21 (14-46)
**GPT**, in [U/L]	
Median (range)	13 (8-24)
**ECOG performance status,** n (%)	
0	0 (0)
1	6 (100)
≥2	0 (0)
**Sites of metastases,** n (%)	
Bone	5 (83.3)
Lymph node	3 (50)
Other	0 (0)
**Prior therapies,** n (%)	
Prostatectomy	4 (66.7)
Radiation	3 (50)
ADT	6 (100)
NAAD	6 (100)
Abiraterone and Enzalutamide	6 (100)
Chemotherapy	6 (100)
Docetaxel only	5 (83.3)
Docetaxel and Cabazitaxel	1 (16.7)
[^223^Ra]Ra-dichloride	4 (66.7)
PSMA-RLT	6 (100)
[^177^Lu]Lu-PSMA-617 RLT	6 (100)
[^225^Ac]Ac-PSMA-617 augmentation	4 (66.7)
Other	5 (83.3)

ADT, antiandrogen deprivation therapy; ALP, alkaline phosphatase; ECOG, Eastern Cooperative Oncology Group; GFR, glomerular filtration rate; GOT, glutamat-oxalacetat-transaminase; GPT, glutamat-pyruvat-transaminase. PSA, prostate specific antigen; NAAD, novel androgen axis drugs; PSMA, prostate specific membrane antigen; RLT, radioligand therapy.

**Table 2:** Monoexponential curve-fitting parameters, time-integrated activity coefficients (TIAC) and mean absorbed dose estimate for [^161^Tb]Tb-PSMA-617 in selected organs. Results are presented as mean values ± standard deviation.

	[^161^Tb]Tb-PSMA-617				[^177^Lu]Lu-PSMA-617
Organ	A (% injected A_0_)	λ (h^-1^)	TIAC (h)	Absorbed dose (mGy/MBq)	Absorbed dose (mGy/MBq)
Kidneys	2.64 ± 1.34	0.021 ± 0.008	1.61 ± 0.55	0.643 ± 0.247	0.545 ± 0.231
Liver	3.72 ± 2.68	0.022 ± 0.008	1.84 ± 1.07	0.148 ± 0.080	0.103 ± 0.057
Parotid gland	0.53 ± 0.30	0.026 ± 0.010	0.18 ± 0.09	0.367 ± 0.198	0.333 ± 0.194
Submand. gland	0.41 ± 0.20	0.027 ± 0.009	0.10 ± 0.05	0.372 ± 0.188	0.285 ± 0.180

**Table 3:** Individual estimated absorbed doses in kidneys, liver, parotid and submandibular glands determined for [^161^Tb]Tb-PSMA-617 vs. [^177^Lu]Lu-PSMA-617

		Absorbed dose (mGy/MBq)							
		[^161^Tb]Tb-PSMA-617				[^177^Lu]Lu-PSMA-617			
Patient	Pre-treatment	Kidneys	Liver	Parotid gland	Subman. gland	Kidneys	Liver	Parotid gland	Subman. Gland
patient 1	N, C, L, R	0.638	0.290	0.793	0.718	0.666	0.199	0.398	0.309
patient 2	N, C, L, A, R	0.537	0.167	0.312	0.223	0.492	0.138	0.150	0.190
patient 3	N, C, L, A, R	0.942	0.196	0.197	0.205	0.906	0.069	0.146	0.112
patient 4	N, C, L, A, R	0.483	0.090	0.338	0.446	0.674	0.125	0.703	0.664
patient 5	N, C, L	0.284	0.051	0.227	0.194	0.252	0.037	0.217	0.178
patient 6	N, C, L, A	0.974	0.093	0.338	0.446	0.278	0.049	0.387	0.259
mean ± SD		0.643 ± 0.247	0.148 ± 0.080	0.367 ± 0.198	0.372 ± 0.188	0.545 ± 0.231	0.103 ± 0.057	0.333 ± 0.194	0.285 ± 0.180

A, [^225^Ac]Ac-PSMA-617 RLT; C, chemotherapy; N, novel androgen axis drugs; L, [^177^Lu]Lu-PSMA-617 RLT; R, [^223^Ra]Ra-dichloride; SD, standard deviation

## Abbreviations

ADT: androgen-deprivation therapy
ALP: alkaline phosphatase
CT: computed tomography
DEW: dual-energy window
ECOG: eastern cooperative oncology group
EMA: European medicines agency
FDA: Food and drug administration
FLAB: fuzzy locally adaptive Bayesian
GFR: glomerular filtration rate
GOT: glutamat-oxalacetat-transaminase
GPT: glutamat-pyruvat-transaminase
HPLC: high-pressure liquid chromatography
ICRP: international commission on radiological protection
LET: linear energy transfer
mCRPC: metastatic castration resistant prostate cancer
MIRD: medical internal radiation dose
NAAD: new androgen axis drugs
OSEM: ordered subset expectation maximization
PCWG: prostate cancer working group
PD: progressive disease
PR: partial regression
PSA: prostate-specific antigen
PSMA: prostate-specific membrane antigen
QC: quality control
RLT: radioligand therapy
ROI: region-of-interest
rTI: relative therapeutic index
SD: stable disease
SPECT: single photon emission computed tomography
SUV_max_: maximum standardized uptake value
TI: therapeutic index
TIA: time-integrated activity
TIAC: time-integrated activity coefficients
VAS: visual analogue scale
VOI: volume-of-interest
