# Supplementary material for: [161Tb]Tb-PSMA-617 radioligand therapy in patients with mCRPC: preliminary dosimetry results and intra-individual head-to-head comparison to [177Lu]Lu-PSMA-617
Source: Theranostics. 2024 Feb 24;14(5):1829–40. doi: 10.7150/thno.92273 (PMC10945337; doi:10.7150/thno.92273)
Supplement: Supplementary file 1 — Supplementary materials and methods, tables. [file thnov14p1829s1.pdf]

SUPPLEMENTAL DATA

Material and Methods

Calibration and recovery correction

To convert count rate to activity for planar scintigraphy, calibration factors were determined based on a vial filled with approx. 50 ml of ^{161}Tb (or ^{177}Lu, respectively) solution with a known activity which was measured during each whole-body scan of the patient. After coregistration of the planar whole body scintigrams, the count rate in a region-of-interest (ROI) surrounding the vial was determined and used to calculate the actual gamma camera response rate for the particular radionuclide in counts/MBq.

To address ring artifacts that were previously published for SPECT imaging of ^{161}Tb [1], an extrinsic uniformity correction map was created using a flood-source phantom filled with aqueous solution of [^{161}Tb]Tb-PSMA-617. The phantom was positioned between the detectors at a distance of approximately 2 cm from each detector surface. A matrix size of 256 x 1024 was applied and a total number of $120*10^6$ counts was acquired for the emission energy of ^{161}Tb (74.6 keV). The acquired map was used for uniformity correction of ^{161}Tb-maging.

To convert the measured voxel values in the reconstructed SPECT images to ^{161}Tb and ^{177}Lu activity, respectively, camera calibration factors were determined [2]. A SPECT/CT scan of a large water cylinder (6595 mL) containing a well calibrated source of the

respective radionuclide (^{161}Tb or ^{177}Lu) was acquired. Here, the same acquisition protocol and reconstruction method were used, which were applied in the corresponding patient studies including all necessary corrections. These measurements were used to derive the calibration factors for both radionuclides in units of Bq/cps.

Recovery coefficients were determined for ^{161}Tb and ^{177}Lu, respectively, by phantom measurements on a NEMA/IEC standard phantom and an in-house phantom, that both enclose fillable glass spheres of different volumes (NEMA-phantom: 1 mL up to 24 mL; in-house phantom: 13 mL up to 63 mL) [3]. Using the same acquisition protocol and reconstruction settings which were applied in the corresponding patient studies, recovery coefficients for ^{161}Tb and ^{177}Lu, respectively, were determined for the different sphere volumes and presented in Table S1. For Recovery correction, the sphere volumes were used that approximate the organ or tumor volumes determined by CT.

Table S1: Recovery coefficients for ^{161}Tb and ^{177}Lu, respectively

**Sphere Volume (mL)**	**^{177}Lu**	**^{161}Tb**
>20	0.80	0.82
13	0.70	0.75
10	0.67	0.70
6	0.59	0.62

Table S2: Monoexponential curve-fitting parameters, time-integrated activity coefficients (TIAC) and mean absorbed dose estimate for [^{177}Lu]Lu-PSMA-617 in selected organs. Results are presented as mean values ± standard deviation.

	[^{177}Lu]Lu-PSMA-617		
**Organ**	**A** (% injected A_0)	**λ** (h^{-1})	**TIAC** (h)
Kidneys	4.80 ± 1.94	0.028 ± 0.011	1.59 ± 0.68
Liver	2.28 ± 0.88	0.030 ± 0.009	1.65 ± 1.36
Parotid gland	0.53 ± 0.20	0.031 ± 0.005	0.13 ± 0.09
Submand. gland	0.41 ± 0.26	0.030 ± 0.007	0.06 ± 0.05

Table S3: Comparison of time-integrated activity coefficients (TIAC) of kidneys and liver with corresponding organ functions assessed by glomerular filtration rate (GFR), creatinine, glutamat-oxalacetat-transaminase (GOT) and glutamat-pyruvat-transaminase (GPT). Results are presented as mean values ± standard deviation.

	**[^{161}Tb]Tb-PSMA-617 RLT**	**[^{177}Lu]Lu-PSMA-617 RLT**
**Kidneys**		
TIAC (h)	1.61 ± 0.55	1.59 ± 0.68
Organ function*		
GFR (mL/min)	58.9 ± 21.9	55.9 ± 18.4
Creatinine (mg/dL)	1.29 ± 0.44	1.28 ± 0.34
**Liver**		
TIAC (h)	1.84 ± 1.07	1.65 ± 1.36
Organ function*		
GOT (U/L)	23.8 ± 12.1	22.0 ± 8.3
GPT (U/L)	14.2 ± 6.4	16.5 ± 6.5

* measured at administration of [^{161}Tb]Tb-PSMA-617 and [^{177}Lu]Lu-PSMA-617

Table S4: Localization and size of lesions based on PSMA-targeted PET/CT

Lesions	Type	Localization	Size (mL)
**patient 1**			
L1	bone	spine	3.0
L2	bone	spine	7.8
L3	bone	pelvic bone	20.0
**patient 2**			
L1	bone	spine	5.2
L2	bone	pelvic bone	30.4
L3	bone	skull	1.7
**patient 3**			
L1	bone	scapula	18.9
L2	bone	scapula	10.5
L3	bone	femur	26.0
**patient 4**			
L1	bone	scapula	43.0
L2	bone	scapula	37.0
L3	bone	femur	28.0
**patient 5**			
L1	lymph node	mediastinum	16.5
L2	lymph node	supraclavicular	6.2
L3	bone	pelvic bone	28.0
**patient 6**			
L1	lymph node	mediastinum	11.0
L2	lymph node	mediastinum	3.7

Table S5: Individual PSA values and change after one cycle [^{161}Tb]Tb-PSMA-617 RLT

	**Pre-treatment**	**PSA at baseline** (ng/mL)	**PSA after [^{161}Tb]Tb-PSMA-617** (ng/mL)	**ΔPSA** (%)	**PCWG3**
**patient 1**	N, C, L, R	474	221	-53.4	PR
**patient 2**	N, C, L, A, R	462	376	-18.6	SD
**patient 3**	N, C, L, A, R	97.6	145	+48.6	PD
**patient 4**	N, C, L, A, R	2148	3721	+73.2	PD
**patient 5**	N, C, L	43.8	51.7	+18.0	SD
**patient 6**	N, C, L, A	72.7	55.1	-24.2	SD

A, [^{225}Ac]Ac-PSMA-617 RLT; C, chemotherapy; N, novel androgen axis drugs; L, [^{177}Lu]Lu-PSMA-617 RLT; PD, progressive disease; PR, partial remission; R, [^{223}Ra]Ra-dichloride; SD, stable disease.

References

1. Marin I, Rydèn T, Van Essen M, Svensson J, Gracheva N, Köster U, et al. Establishment of a clinical SPECT/CT protocol for imaging of ^{161}Tb. EJNMMI Phys. 2020; 7: 45.

2. Ljungberg M, Celler A, Konijnenberg MW, Eckerman KF, Dewaraja YK, Sjögreen-Gleisner K, et al. MIRD Pamphlet No. 26: Joint EANM/MIRD guidelines for quantitative ^{177}Lu SPECT applied for dosimetry of radiopharmaceutical therapy. J Nucl Med. 2016; 57: 151–62.

3. Schaefer A, Kremp S, Hellwig D, Rübe C, Kirsch C-M, Nestle U. A contrast-oriented algorithm for FDG-PET-based delineation of tumour volumes for the radiotherapy of lung cancer: derivation from phantom measurements and validation in patient data. Eur J Nucl Med Mol Imaging. 2008; 35: 1989–99.
